# Literary Notes

**Published:** 1906-07

**Authors:** 


### LITERARY NOTES.

MESSRS. P. Blakiston's Son and Company, Philadelphia, have issued a new medical catalogue called "The Illustrated." It is a sixteen page quarto with covers, and contains valuable information concerning the latest medical works. This famous house has been established sixty-three years and enjoys the patronage of purchasers in all parts of the world. A catalogue will be furnished on application. Address the publishers, 1012 Walnut Street.

The Cleveland Press, Chicago, has issued an announcement of early forthcoming medical works, among which is one by Alexander Hugh Ferguson, entitled, The technic of modern operations for hernia. Many other interesting volumes are mentioned and the announcement is illustrated.

